# Colon-derived Caco-2 cells support replication of hepatitis E virus genotype 1 strain Sar55 generated by reverse genetics

**DOI:** 10.1016/j.virusres.2024.199427

**Published:** 2024-06-27

**Authors:** Alexander Falkenhagen, Jessica Panajotov, Reimar Johne

**Affiliations:** Department of Biological Safety, German Federal Institute for Risk Assessment, Max-Dohrn-Str. 8-10, 10589 Berlin, Germany

**Keywords:** Hepatitis E virus, Cell culture, Reverse genetics, Sar55, Genotype 1, Intestine

## Abstract

The hepatitis E virus (HEV) is infecting over 20 million people annually with a high morbidity especially in pregnant women and immune-suppressed individuals. While HEV genotype 1 (HEV-1) infects only humans, genotype 3 (HEV-3) is zoonotic and commonly transmitted from infected animals to humans. Whereas a few reverse genetics systems enabling targeted genome manipulations exist for HEV-3, those for HEV-1 are still very limited, mainly because of inefficient cell culture replication. Here, the generation of HEV-1 strain Sar55 and HEV-3 strain 47832mc by transfecting *in vitro*-transcribed and capped virus genomes into different cell lines was attempted. Culture supernatants of colon-derived colorectal adenocarcinoma cell line Caco-2 contained HEV-1 and HEV-3 capable of infecting Caco-2 cells. Density gradient centrifugation analyses of culture supernatants confirmed that HEV-1 particles were quasi-enveloped in analogy to HEV-3 and that non-virion-associated capsid protein was secreted from cells. Following transfection or infection of Caco-2 cells, HEV-1 consistently reached higher titers than HEV-3 in culture supernatants, but HEV-1 generated by transfection of Caco-2 cells was unable to efficiently infect hepatoma cell lines PLC/PRF/5 or HuH7-Lunet BLR. Taken together, our results indicate that HEV-1 is able to exert a complete replication cycle in Caco-2 cells. An efficient cell culture system for this genotype will be useful for studying species tropism, but further research is required to determine the significance of HEV-1 replication in colon-derived cells.

## Highlights

• Lack of efficient HEV-1 cell culture systems hinders research.
• Comparison of revere genetics approaches for the generation of HEV-1 and HEV-3.
• HEV-1 strain Sar55 replicates efficiently in colon-derived Caco-2 cells.
• Robust reverse genetics system enables investigation of many aspects of HEV-1.

## 1 Introduction

The hepatitis E virus (HEV) is the causative agent of hepatitis E, an inflammation of the liver that is mostly acute and transient, but can become chronic in immunosuppressed patients. The World Health Organization estimates that over 20 million HEV infections occur every year (WHO, 2023). While the majority of infections are clinically silent or cause only mild symptoms, HEV infections can also lead to fulminant hepatic failure (Wedemeyer et al., 2012). Mortality rates range from 0.5% to 4% in the general population, but can be significantly higher in pregnant women, patients with pre-existing liver disease, and immunocompromised individuals (Johne et al., 2022). There is also increasing evidence that HEV infection is associated with extrahepatic manifestations including neurological and kidney disorders (Jha et al., 2021; Velavan et al., 2021).

HEV is classified as a member of the species *Paslahepevirus balayani* and comprises eight different genotypes that are referred to as HEV-1 to HEV-8 (Purdy et al., 2022). These genotypes differ in their geographic distribution, host range, and mode of transmission. The majority of human hepatitis E cases are caused by HEV-1 to HEV-4. HEV-1 and HEV-2 only infect humans and other higher primates. Human-to-human transmission of these genotypes occurs via the fecal-oral route and large outbreaks due to contaminated drinking water have been described in countries with lower hygiene standards (Nelson et al., 2019; Pallerla et al., 2020). In contrast, HEV-3 and HEV-4 are zoonotic pathogens that primarily spread to humans by the consumption of raw or undercooked meat products from infected animals, such as domestic pigs or wild boars (Yugo and Meng, 2013).

HEV particles in fecal samples from infected individuals are pre-dominantly non-enveloped (Balayan et al., 1983). However, virions in the blood of patients as well as in the supernatant of infected cell cultures have a quasi-envelope (Takahashi et al., 2010; Takahashi et al., 2008). The HEV genome consists of one single-stranded RNA molecule of positive polarity. It is approximately 7.200 nucleotides in length and contains a 5’ cap, a 5’ untranslated region (UTR), three to four open reading frames (ORF1-4), a 3’UTR and a polyA tail. ORF1 encodes a polyprotein that contains multiple functional domains including the RNA-dependent RNA polymerase (RdRp) and a hypervariable region (HVR) that can harbor insertions of cellular or viral origin (Johne et al., 2014; Shukla et al., 2012). ORF2 and ORF3 are translated from a subgenomic viral mRNA that is transcribed from the negative-strand RNA genome using the RdRp (Graff et al., 2006). ORF2 encodes the capsid protein and contains two start codons (Yin et al., 2018). Translation from the first start codon leads to a glycosylated form of the capsid protein that is secreted from infected cells and not associated with viral genomes, while translation initiation from the second start codon results in the virion-forming capsid protein. The ORF2 capsid protein is highly immunogenic, but is masked in quasi-enveloped HEV particles (Takahashi et al., 2010). The ORF3 protein is a small multifunctional protein that can modulate cellular pathways, but is also associated with the HEV quasi-envelope and plays a role in virion release from infected cells (Sari et al., 2021). ORF4 is only present in HEV-1 and was shown to increase the activity of the RdRp (Nair et al., 2016).

For elucidation of determinants of host specificity, experiments using reverse genetics (RG) enabling targeted genome manipulations are tremendously helpful. To date, RG systems for all known HEV genotypes with the exception of HEV-2 have been developed (Primadharsini et al., 2024; Scholz et al., 2020b; Zhang et al., 2021). The majority of these RG systems are based on transfecting *in vitro*-transcribed and capped HEV RNA genomes into susceptible cell lines, which should result in the release of infectious virus. However, RG systems for HEV-1 are still limited, mainly because this genotype generally replicates poorly in cell cultures. One of the most utilized HEV-1 strains is Sar55 (HEV-1_Sar55_), which was identified in a patient during an outbreak in Pakistan (Iqbal et al., 1989). Cynomolgus monkeys intravenously inoculated with stool suspension from that patient became infected and the entire genome could be sequenced (Tsarev et al., 1992). To this date, fecal suspensions from monkeys are used for HEV-1 studies (Behrendt et al., 2022), but the strain could never be fully adapted to cell culture (Shukla et al., 2012). In order to establish an RG system for HEV-1_Sar55_, Emerson et al. transfected capped viral genomes into various cell lines and showed that infectious virus was present in lysates of transfected hepatoma cells (Emerson et al., 2004; Emerson et al., 2006). However, little to no infectious virus was released from transfected or infected cells and virus spread was not observed in cell culture. In a follow-up study, transfected human colon-derived colorectal adenocarcinoma Caco-2 cells and the Caco-2 subclone C25j released HEV-1_Sar55_ particles into the culture media that could infect another hepatoma cell line, HepG2/C3A (Emerson et al., 2010), but these results have not been confirmed elsewhere. Indeed, a recent study has used HuH7-S10-3 cell lysate-derived HEV-1_Sar55_ to infect HepG2/C3A cells, but no HEV-positive foci were detected, suggesting that efficient infection of HepG2/C3A cells with HEV-1_Sar55_ is still debatable (Wang et al., 2022). In the absence of an efficient RG system that allowed to examine HEV-1_Sar55_ spread and replication in cell culture, replicon systems and animal experiments were previously used to analyze the impact of mutations on HEV-1_Sar55_ (Cordoba et al., 2012; Feagins et al., 2011; Tian et al., 2020; Wang et al., 2022).

In contrast, multiple HEV-3 strains have been adapted to replication in cell culture. HEV-3 strain 47832 was originally identified in a chronically infected transplant patient and contains an insertion within the HVR derived from viral genome duplications (Johne et al., 2014). Following passaging of virus contained in a serum sample using the human lung carcinoma cell line A549, multiple point mutation were detected across the viral genome and this cell culture-adapted virus was designated 47832c (HEV-3_47832c_)(Johne et al., 2014). Later, a plasmid-based RG system for the generation of a molecular clone of cell culture-adapted HEV-3_47832c_, referred to as 47832mc (HEV-3_47832mc_), has been developed (Scholz et al., 2020a; Scholz et al., 2021). The amino acid sequence of HEV-3_47832mc_ is identical to HEV-3_47832c_, but HEV-3_47832mc_ contains multiple synonymous mutations in comparison to HEV-3_47832c_.

In order to improve the RG system for HEV-1_Sar55_, transfection of *in vitro*-transcribed and capped RNA was systematically analyzed here in different human cell lines. HEV-3_47832mc_ was used as control and for comparison. HEV-1_Sar55_ and HEV-3_47832mc_ replication were evident after culture supernatants of transfected cells were used to inoculate colon-derived Caco-2 cells. HEV-1_Sar55_ replicated to high titers in Caco-2 cells and could be characterized further. The results indicate that HEV-1_Sar55_ is able to exert a complete replication cycle in Caco-2 cells. An efficient cell culture system for this genotype will be useful for studying many aspects of HEV basic and applied research including factors determining species tropism, replication kinetics and drug resistance.

## 2 Materials and methods

### 2.1 Cell lines and plasmids

PLC/PRF/5 and Caco-2 cells were obtained from the American Tissue Culture Collection (Manassas, VA, USA). HuH7-Lunet BLR cells were kindly provided by Dr. Mathias Schemmerer (University Medical Center Regensburg, Germany). The generation of the A549 cell subclone D3 was described previously (Schemmerer et al., 2016). All cell culture reagents were obtained from PAN-Biotech GmbH (Berlin, Germany). All cell lines were cultured in cMEM (MEM Eagle with Earl's balanced salt solution and 2.2 g/l sodium bicarbonate supplemented with 10% heat-inactivated FBS premium, 2 mM L-glutamine, 1x non-essential amino acid solution, 10 units/mL penicillin and 100 µg/mL streptomycin) and maintained at 37°C and 5% CO_2_. Following transfections and infections, media for PLC/PRF/5 and HuH7-Lunet BLR cells were switched from cMEM to cMEM supplemented with 2.5 µg/mL amphotericin B and 30 mM magnesium chloride, which was previously shown to increase HEV-3 replication in those cell types (Schemmerer et al., 2019). A549/D3 and Caco-2 cells were kept in cMEM. The generation of the plasmid encoding HEV-3_47832mc_ (p47832mc) was described previously (Scholz et al., 2020a). The plasmid encoding HEV-1_Sar55_ (pSK-HEV-2) was kindly provided by Dr. Patrizia Farci (National Institute of Allergy and Infectious Diseases, Bethesda, MD, USA) (Emerson et al., 2001). The identity of the plasmid was confirmed using restriction enzyme digests and sequencing of the HVR to confirm the absence of insertions. Sequencing primers are available upon request.

### 2.2 In vitro RNA synthesis and capping

The plasmids p47832mc and pSK-HEV-2 were linearized by restriction digest and cleaned up using the Monarch PCR & DNA Cleanup Kit (New England Biolabs GmbH, Frankfurt am Main, Germany). *In vitro* RNA was transcribed with the MEGAscript T7 Transcription Kit (Thermo Fisher Scientific, Waltham, MA, USA), cleaned up by lithium chloride precipitation, and capped using the Vaccinia virus capping enzyme system together with mRNA cap 2´-O-methyltransferase (all New England Biolabs GmbH) followed by an additional lithium chloride precipitation.

### 2.3 Transfection of cell cultures

A549/D3 cells were seeded at a density of 6 × 10^5^ cells per well in 6-well plates one day prior to transfection, while all other cell lines were seeded at a density of 2 × 10^5^ cells. A549/D3, PLC/PRF/5, HuH7-Lunet BLR and Caco-2 cells were transfected with 1 µg RNA using the TransIT-mRNA Transfection Kit (Mirus Bio LLC, Madison, WI, USA). At day 1, day 2, and day 3 post-transfection, cell culture media were completely changed. After the media change at day 3, transfected cells were transferred to 34.5°C. Thereafter, media were completely changed every 3-4 days. Collected culture supernatants were clarified by centrifugation at 6000x*g* for 5 min and stored at -80°C before they were analyzed further. Cell lysates were collected by washing transfected cells twice with PBS followed by trypsination and two additional PBS wash steps. The washed cells were resuspended in 400 µl cMEM followed by three freezing and thawing cycles at -80°C and room temperature, respectively. The lysates were clarified by centrifugation at 16,000x*g* for 10 min and stored at -80°C.

### 2.4 Immunofluorescence microscopy and HEV antigen ELISA

ORF2 expression in transfected or infected cells was visualized using an HEV ORF2 protein-specific rabbit hyperimmune serum (kindly provided by Prof. Rainer Ulrich, Friedrich Loeffler Institute, Germany) and a fluorescein isothiocyanate-conjugated anti-rabbit IgG (Sigma, Deisenhofen, Germany) as described previously (Johne et al., 2016). Roti-Mount Fluor Care DAPI (Carl Roth, Karlsruhe, Germany) was used to mount the cells and stain nuclei. Fluorescence analyses were performed using an Axio Observer Z1 microscope (Carl Zeiss AG, Oberkochen, Germany). In order to test for the presence of ORF2 protein in cell culture supernatants and density gradient fractions, HEV Ag ELISA (Wantai BioPharm, Beijing, China) was performed.

### 2.5 RNA extractions and RT-qPCR

RNA was extracted from cell culture supernatants or density gradient fractions using the EMAG Nucleic Acid Extraction System (biomerieux Deutschland GmbH, Nuertingen, Germany). RNA copies/mL were determined by performing RT-qPCR as described previously (Jothikumar et al., 2006) using *in vitro*-transcribed full-length HEV RNA genomes generated as described above as a standard.

### 2.6 Density gradient centrifugation

For density gradient centrifugation, sucrose solutions were prepared in PBS. For analyses of culture supernatants, 1.5 mL 60% sucrose, 1.0 mL 50% sucrose, 1.0 mL 40% sucrose, 1.0 mL 30% sucrose, 1.0 mL 20% sucrose, 1.5 mL 10% sucrose and 1.0 mL cell culture supernatant from transfected cells were layered on top of each other in an open-top ultracentrifuge tube (Beckman Coulter GmbH, Krefeld, Germany). For analysis of cell lysate, 1.5 mL 70% sucrose, 1.0 mL 60% sucrose, 1.0 mL 50% sucrose, 1.0 mL 40% sucrose, 1.0 mL 30% sucrose, 1.0 mL 20% sucrose and 1.0 mL 10% sucrose were layered on top of each other before 5 mL cMEM containing 1 × 10^7^ HEV-1_Sar55_ genome copies were added. Tubes were loaded into an SW 41 Ti Swinging-Bucket Rotor (Beckman Coulter GmbH) and centrifuged at 150,000x*g* and 4°C in an Optima LE-80 ultracentrifuge (Beckman Coulter GmbH) for 18h. After centrifugation, 1.0 mL fractions were collected from the top and density was determined using the DR201-95 digital refractometer (Kruess GmbH, Hamburg, Germany) before the fractions were frozen at -80°C.

### 2.7 Infection of cell cultures and ribavirin treatment

For infections in 6-well plates, 2 × 10^5^ cells were seeded per well. After incubation for 1 day, cells were washed twice with PBS and virus in cMEM was added (1 mL total volume). The cells were incubated for 1.5 h at room temperature before 1 mL of fresh cMEM was added and the cells were transferred to 34.5°C and 5% CO_2_. Cell culture media were completely exchanged with fresh cMEM on day 1, day 2, and day 3 post-infection. Thereafter, media were completely exchanged every 3-4 days until the end of the experiment. Where indicated, infected cells were washed five times with PBS to remove the virus inoculum on day 1 post-infection before fresh media were added. Thereafter, media were completely exchanged every 1-4 days. Where indicated, ribavirin (Merck, Darmstadt, Germany) was added to the inoculum and culture media (final concentration 10 µM). Cell culture supernatants were collected at the indicated days, clarified by centrifugation at 6,000x*g* for 5 min, and frozen at -80°C prior to analyses. For infections in 96-well plates, 1 × 10^4^ cells were seeded per well. One day later, cells were washed twice with PBS and infected with serial virus dilutions (100 µl total volume). After 1.5 h at room temperature, 100 µl of fresh culture media were added and the cells were transferred to 34.5°C and 5% CO_2_. Media were completely exchanged with 100 µl fresh culture media every 3-4 days until the cells were analyzed by immunofluorescence microscopy to determine the number of focus forming units (FFU)/mL 21 days post-infection.

### 2.8 Statistical analysis

Data are presented as single data points and/or mean +/- standard deviation. Statistical significance was determined using a two-tailed unpaired t test. Results with a p-value equal to or below 0.05, 0.01, or 0.001 were considered statistically significant and marked with one, two, or three asterisks, respectively.

## 3 Results

### 3.1 Transfected hepatoma and colorectal adenocarcinoma cells express HEV ORF2

**Fig. 1:**
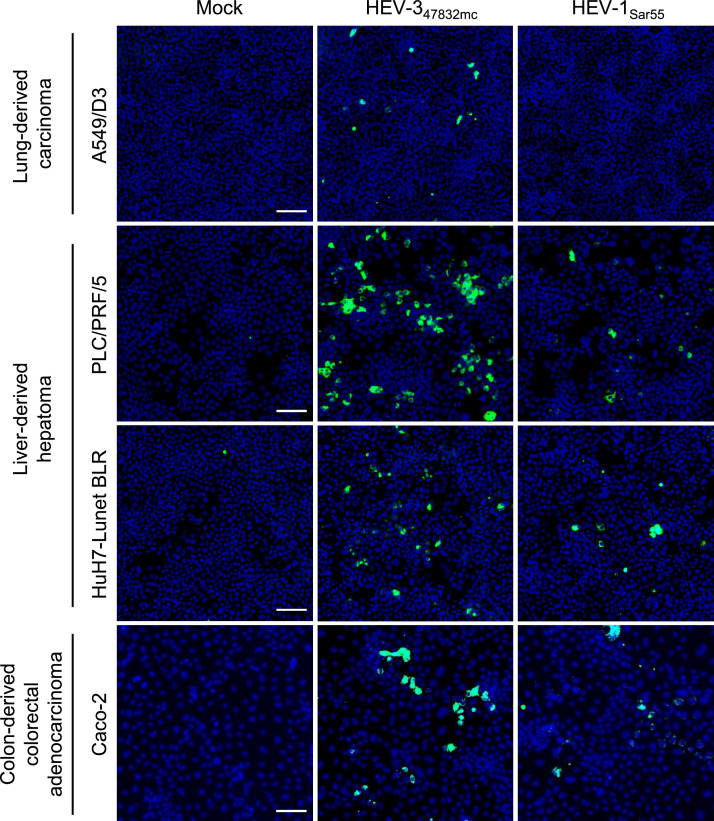
Transfection of genomic HEV RNA results in detectable HEV-1_Sar55_ and HEV-3_47832mc_ ORF2 expression in PLC/PRF/5, HuH7-Lunet BLR, and Caco-2 cells. The indicated cell lines were transfected with HEV RNA and ORF2 expression was visualized by an immunofluorescence assay 7 days post-transfection. Data are representative of two similar experiments performed independently. Scale bar = 100 µm.

In order to improve the RG system for HEV-1_Sar55_, we decided to utilize *in vitro*-transcribed capped HEV-1_Sar55_ and HEV-3_47832mc_ RNA genomes to transfect different human cell lines. We chose the human lung-derived carcinoma cell line subclone A549/D3, the human liver-derived hepatoma cell lines PLC/PRF/5 and HuH7-Lunet BLR, as well as the colon-derived colorectal adenocarcinoma cell line Caco-2. Following transfection with HEV-3_47832mc_ RNA genomes, ORF2 expression was evident in all four tested cell lines (Fig. 1) as determined by immunofluorescence microscopy 7 days post-transfection. In contrast, transfection with HEV-1_Sar55_ RNA genomes only resulted in ORF2 protein-positive PLC/PRF/5, HuH7-Lunet BLR, and Caco-2 cells, but no ORF2 expression was detected in A549/D3 cells (Fig. 1). While the presence or absence of ORF2 expression alone is not sufficient to conclude whether HEV replicates, the results suggested that HEV-1_Sar55_ replication was less efficient in A549/D3 cells than in the other cell types under the tested conditions. Therefore, we decided to further compare the generation of HEV-1_Sar55_ and HEV-3_47832mc_ in PLC/PRF/5, HuH7-Lunet BLR, and Caco-2 cells.

### 3.2 Culture supernatants of transfected Caco-2 cells contain HEV-1_Sar55_ and HEV-3_47832mc_ capable of infecting Caco-2 cells

**Fig. 2:**
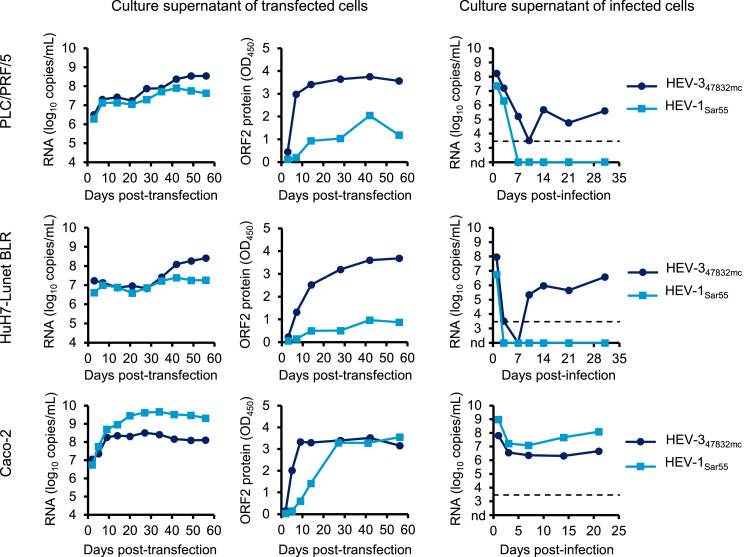
Transfection of HEV RNA genomes into Caco-2 cells leads to release of HEV-1_Sar55_ capable of infecting Caco-2 cells. The indicated cell lines were transfected with HEV RNA and culture supernatants were analyzed for the presence of HEV genomes by RT-qPCR and HEV ORF2 protein by ELISA. Culture supernatants from transfected cells were used to inoculate the respective cell line and culture supernatants were monitored for HEV RNA by RT-qPCR. In these experiments, the virus inoculum was not removed by washing, resulting in high HEV RNA titers in the beginning of the experiment. Due to their duration, the experiments presented in this figure were only performed once.

PLC/PRF/5, HuH7-Lunet-BLR, and Caco-2 cells were transfected with HEV-1_Sar55_ or HEV-3_47832mc_ RNA genomes and culture media were completely exchanged with fresh media every three to four days until day 56 post-transfection. Cell culture supernatants were analyzed for the presence of viral genomes by RT-qPCR (Fig. 2). Following transfection, genome copies increased over time for both HEV-1_Sar55_ and HEV-3_47832mc_ in all tested cell lines. In culture supernatants of transfected hepatoma cell lines PLC/PRF/5 and HuH7-Lunet BLR, higher numbers of viral genomes were consistently observed for HEV-3_47832mc_ in comparison to HEV-1_Sar55_. In contrast, culture supernatants of transfected colorectal adenocarcinoma cell line Caco-2 consistently contained higher numbers of HEV-1_Sar55_ genomes. The same culture supernatants were also analyzed for the presence of ORF2 protein using a commercial HEV antigen ELISA. For both HEV-1_Sar55_ and HEV-3_47832mc_, increasing quantities of ORF2 protein were detected in culture supernatants of all transfected cell lines (Fig. 2). Irrespective of the transfected cell line, higher ORF2 protein quantities were detected for HEV-3_47832mc_ in comparison to HEV-1_Sar55_ especially in the first weeks after transfection, showing that there was a lack of correlation between the HEV RNA titers and the detected ORF2 antigen levels in Caco-2 cells.

At the end of the experiment on day 56 post-transfection, culture supernatants contained ∼7.5 log_10_ HEV-1_Sar55_ and ∼8.5 log_10_ HEV-3_47832mc_ copies/mL (PLC/PRF/5), ∼7.0 log_10_ HEV-1_Sar55_ and ∼8.5 log_10_ HEV-3_47832mc_ copies/mL (HuH7-Lunet BLR), and ∼9.0 log_10_ HEV-1_Sar55_ and ∼8.0 log_10_ HEV-3_47832mc_ copies/mL (Caco-2). In order to determine if infectious virus was present in culture supernatants of transfected cells, the culture supernatants from the respective transfected cell lines were used to inoculate PLC/PRF/5, HuH7-Lunet BLR or Caco-2 cells. To minimize the bottleneck that infection of specific cell lines represents, the near maximal volume of culture supernatant (1 mL) was used for inoculation irrespective of the number of genome copies present. The culture supernatants of the inoculated cells were completely exchanged with fresh media every 3-4 days and the number of RNA genome copies/mL in collected culture supernatants was determined by RT-qPCR (Fig. 2). HEV-3_47832mc_ replication was evident after PLC/PRF/5, HuH7-Lunet BLR or Caco-2 cells were inoculated with supernatants from the respective transfected cell line (Fig. 2). However, HEV-1_Sar55_ replication was only observed after inoculation of Caco-2 cells with culture supernatants from transfected Caco-2 cells (Fig. 2). Therefore, we chose to further characterize HEV-1_Sar55_ generation and replication in Caco-2 cells.

### 3.3 HEV-1_Sar55_ can consistently be generated and replicates more efficiently than HEV-3_47832mc_ in Caco-2 cells

**Fig. 3:**
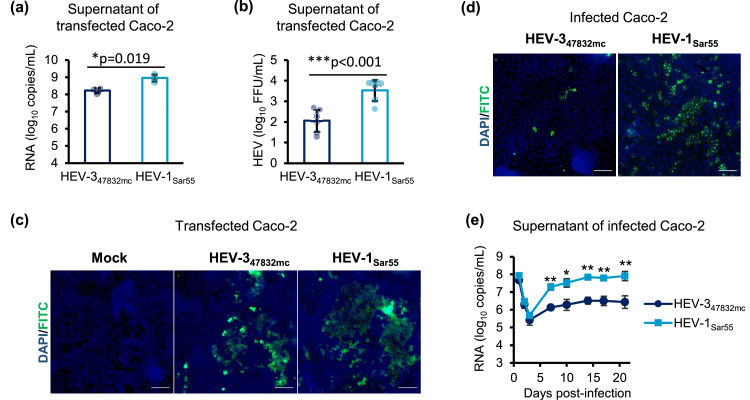
HEV-1_Sar55_ replicates more efficiently than HEV-3_47832mc_ in Caco-2 cells. **(a and b)** Caco-2 cells were transfected with HEV RNA genomes and culture supernatants were collected 14 days post-transfection. **(a)** The number of RNA genome copies/mL was determined by RT-qPCR. Data are means +/- SD and representative of three independent transfections. **(b)** The number of FFU/mL was determined by infecting Caco-2 seeded in 96-well plates followed by immunofluorescence microscopy. Data are means +/- SD and representative of three independent experiments with titrations performed in duplicates. **(c)** Immunofluorescence microscope images of transfected Caco-2 cells 14 days-post-transfection. **(d)** Immunofluorescence microscope images of Caco-2 cells seeded in 96-well plates and infected with 100 µl culture supernatant from transfected Caco-2 cells 21 days post-infection. **(e)** Caco-2 cells seeded in 6-well plates were infected with 8 log_10_ HEV RNA genome copies and the number of RNA genomes released into the culture media was monitored by RT-qPCR. In these experiments, the virus inoculum was not removed by washing, resulting in high HEV RNA titers in the beginning of the experiment. Data are means +/- SD and representative of three independent infections. Scale bar = 100 µm; *p≤0.05; **p≤0.01; ***p≤0.001.

To confirm that virus generation using Caco-2 cells was consistent, transfections were repeated with HEV-1_Sar55_ and HEV-3_47832mc_ RNA genomes. Culture supernatants were collected 14 days post-transfection and analyzed for the presence of viral genomes by RT-qPCR as well as for the infectious titer expressed in FFU/mL by immunofluorescence microscopy of infected Caco-2 cells. Higher numbers of genome copies/mL (Fig. 3a) and FFU/mL (Fig. 3b) were consistently detected for HEV-1_Sar55_ in comparison to HEV-3_47832mc_. ORF2 expression was detected in both transfected (Fig. 3c) and infected cells (Fig. 3d). Following infection of Caco-2 cells with an equal number of HEV-1_Sar55_ and HEV-3_47832mc_ genome copies, the HEV RNA titer in supernatans was determined using RT-qPCR. Consistently higher HEV-1_Sar55_ RNA genome titers were observed starting at day 7 post-infection until the end of the experiment at day 21 post-infection (Fig. 3e).

### 3.4 Caco-2 cells release quasi-enveloped HEV-1_Sar55_ and ORF2 protein not associated with virions

**Fig. 4:**
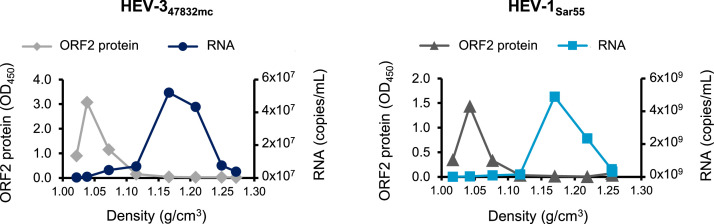
Caco-2 cells release quasi-enveloped HEV-1_Sar55_ and ORF2 not associated with virions into culture supernatants. One mL of culture supernatants from transfected Caco-2 cells containing ∼9.5 log_10_ HEV-1_Sar55_ or ∼8.0 log_10_ HEV-3_47832mc_ copies was analyzed by density gradient centrifugation. The density of the different fractions was determined and the genome copy number as well as ORF2 antigen levels in the different fractions were analyzed by RT-qPCR and ELISA, respectively. Data are representative of two similar experiments performed independently.

The majority of intracellular HEV in cell lysates of infected cells is non-enveloped, but HEV gains a quasi-envelope during egress from infected cells without apparent cytopathic effects. Therefore, extracellular HEV in culture supernatants and patient serum is mostly quasi-enveloped. In infected individuals, it is assumed that HEV loses the quasi-envelope by passing through the bile duct before being excreted in feces. However, Emerson et al. reported that HEV-1_Sar55_ in feces of an infected rhesus monkey and HEV-1_Sar55_ in culture supernatants of transfected cells was associated with lipids (Emerson et al., 2010). To confirm that HEV-1_Sar55_ is released from transfected Caco-2 cells with a quasi-envelope, we performed density gradient centrifugation, which is commonly used to separate non-enveloped virus particles that have a higher density from quasi-enveloped virus particles which have a lower density. One mL of culture supernatant of Caco-2 cells was layered on top of a sucrose gradient. Following utracentrifugation, fractions were collected and analyzed for the presence of viral genomes by RT-qPCR. The majority of HEV-1_Sar55_ and HEV-3_47832mc_ genomes banded at a density of 1.17 g/cm^3^, which was similar to previously reported densities for quasi-enveloped HEV-3 particles in comparable experiments (Fig. 4) (Nagashima et al., 2013; Takahashi et al., 2010).

Yin et al. have shown that translation initiation from the first ORF2 start codon resulted in the secretion of high quantities of non-virion-associated ORF2 protein in hepatoma cell lines (Yin et al., 2018). To examine if colon-derived Caco-2 cells also secrete non-virion associated ORF2 protein, we analyzed the same fractions from the density gradient centrifugation by ORF2 protein ELISA. The results showed that the majority of HEV-1_Sar55_ and HEV-3_47832mc_ ORF2 protein banded at a much lower density than the viral genomes (Fig. 4), indicating that the detected ORF2 protein was indeed not associated with virions.

### 3.5 Caco-2 cells, but not hepatoma cell lines, are sensitive to HEV-1_Sar55_ infection

**Fig. 5:**
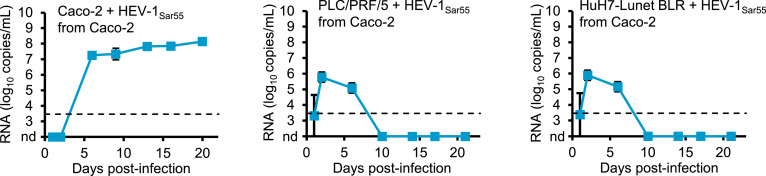
Caco-2-derived HEV-1_Sar55_ replicates inefficiently in hepatoma cell lines PLC/PRF/5 and HuH7-Lunet BLR. **(a)** Caco-2, PLC/PRF/5, and HuH7-Lunet BLR cells seeded in 6-well plates were inoculated with 8 log_10_ HEV-1_Sar55_ RNA genome copies, washed to remove the virus inoculum, and the number of RNA genome copies/mL in collected culture supernatants was detected by RT-qPCR. Data are means +/- SD and representative of two independent infections.

In order to determine whether HEV-1_Sar55_ generated by transfection of Caco-2 cells can infect hepatoma cell lines, Caco-2, PLC/PRF/5 and HuH7-Lunet BLR cells were inoculated with culture supernatants from transfected Caco-2 cells containing the same number of HEV-1_Sar55_ RNA copies. For these experiments, the cells were washed to remove unbound virus after inoculation and the number of viral RNA genomes released into culture supernatants was monitored by RT-qPCR. While RNA copies increased in inoculated Caco-2 cells over time, we observed a short peak of viral RNA in inoculated PLC/PRF/5 and HuH7-Lunet BLR cells followed by a decline to undetectable levels, suggesting that infection of hepatoma cell lines was inefficient under the tested conditions (Fig. 5).

To confirm that HEV-1_Sar55_ titer increases detected in the supernatant of Caco-2 cells were due to virus replication, infections were also performed in the presence or absence of ribavirin, a guanosine nucleoside analogue that inhibits viral RNA synthesis. Ribavirin was used at a concentration of 10 µM, which has previously been shown to result in partial inhibition of an HEV-1_Sar55_ replicon system (Wang et al., 2022). In the presence of ribavirin, 1-2 log_10_ lower RNA titers were observed from 7 days post-infection until 21 days post-infection (Supplemental Fig. S1a). In the absence of ribavirin, clear ORF2 protein-positive foci were detected by immunofluorescence microscopy at the end of the experiment, while no comparable foci were detected in infected cells treated with ribavirin (Supplemental Fig. S1b).

**Fig. 6:**
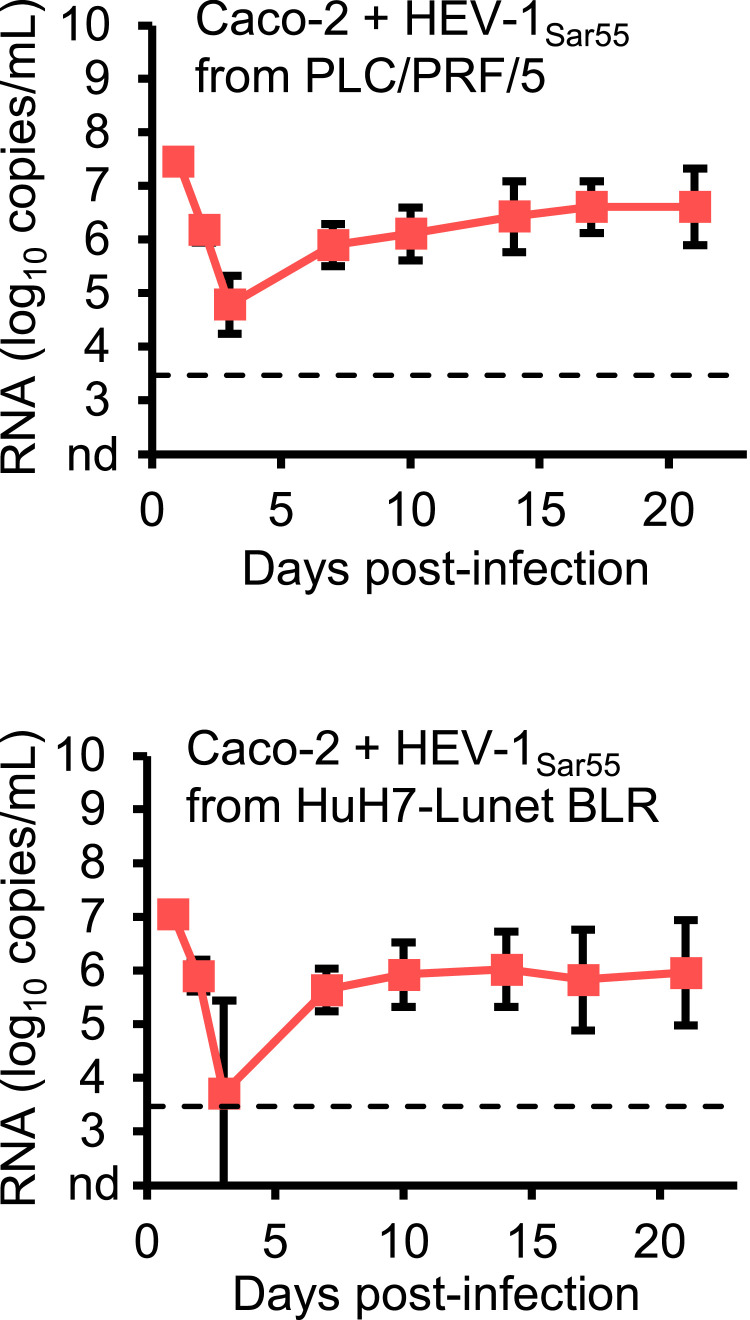
PLC/PRF/5- and HuH7-Lunet BLR-derived HEV-1_Sar55_ replicates in Caco-2 cells. Caco-2 cells seeded in 6-well plates were inoculated with culture supernatants of transfected PLC/PRF/5 or HuH7-Lunet BLR cells containing 7.5 log_10_ or 7.0 log_10_ HEV-1_Sar55_ genome copies, respectively, and the number of RNA genome copies/mL was monitored by RT-qPCR. In these experiments, the virus inoculum was not removed by washing, resulting in high HEV RNA titers in the beginning of the experiment. Data are means +/- SD and representative of two independent infections.

As Caco-2 cells were sensitive to HEV-1_Sar55_ infection, we also inoculated Caco-2 cells with culture supernatants of transfected PLC/PRF/5 or HuH7-Lunet BLR cells containing comparable numbers of HEV-1_Sar55_ genome copies as were used for hepatoma cell line infection experiments shown in Fig. 2 (∼7.5 log_10_ and ∼7.0 log_10_ RNA copies for PLC/PRF/5 and HuH7-Lunet BLR cells, respectively). Interestingly, we detected HEV-1_Sar55_ replication in Caco-2 cells inoculated with culture supernatants of either transfected PLC/PRF/5 or HuH7-Lunet BLR cells (Fig. 6), suggesting that the culture supernatant of these transfected cells contained infectious HEV-1_Sar55_.

It has previously been shown that non-enveloped HEV-3 in cell lysates of hepatoma cell lines was more infectious for hepatoma cell line HepG2/C3 than quasi-enveloped HEV-3 in culture supernatants (Yin et al., 2016). To characterize non-enveloped HEV-1_Sar55_, we transfected Caco-2 cells and collected culture supernatants as well as cell lysates 14 days post-transfection. Cell lysates contained 1-2 log_10_ less HEV RNA copies and FFU (Supplemental Fig. S2a) than culture supernatants. Density gradient centrifugation of cell lysates showed that virus genome copies peaked at a density of 1.27 g/cm^3^ (Supplemental Fig. S2b), which was similar to the reported sucrose density of HEV contained in feces (Takahashi et al., 2010). However, genome copies were also detected at lower densities, suggesting that virus in cell lysates was a mixed population of quasi-enveloped and non-enveloped particles. Following infection of Caco-2 cells with an equal number of HEV-1_Sar55_ genomes derived from culture supernatants or cell lysates, comparable numbers of genome copies were released 14 days post-infection (Supplemental Fig. S2c). Taken together, these results indicated that cell lysate-derived HEV-1_Sar55_ was not more infectious for Caco-2 cells than culture supernatant-derived virus.

## 4 Discussion

Investigation of HEV-1 and HEV-3 differences, e.g. regarding the determinants of their different host range, is currently hindered by the poor replication of HEV-1 in cell culture. We have compared different RG approaches for the generation of HEV-1_Sar55_ and HEV-3_47832mc_ and shown that human colon-derived colorectal adenocarcinoma Caco-2 cells support the generation and replication of both genotypes, whereby higher titers for HEV-1_Sar55_ were observed. An efficient RG and cell culture system for HEV-1_Sar55_ will be useful for studying many aspects of HEV basic and applied research.

The hepatoma cell lines PLC/PRF/5 cells and HuH7-Lunet BLR have previously been shown to support HEV-3_47832mc_ replication (Schemmerer et al., 2016; Schemmerer et al., 2019), but have not been used as a transfection host for the generation of HEV-3_47832mc_ by reverse genetics. Our results show that both cell lines are suitable transfection hosts for the generation of HEV-3_47832mc_. In contrast, HEV-1_Sar55_ replicated less efficiently in these two cell lines under the tested conditions. While HuH7-Lunet BLR cells have previously not been tested for the generation of HEV-1_Sar55_, these results are in line with previous reports of poor spread of HEV-1_Sar55_ in hepatoma cell lines, such as PLC/PRF/5 or HuH7 cells (Emerson et al., 2004; Emerson et al., 2006).

Following transfection of lung-derived carcinoma cell line subclone A549/D3, no HEV-1_Sar55_ ORF2 expression was observed, suggesting limited replication under the tested conditions in the utilized cells. A549 cells and their subclones have previously been reported to support replication of other HEV-1 strains (Huang et al., 1999; Huang et al., 1995; Primadharsini et al., 2023; Takahashi et al., 2010), but only very recently Primadharsini et al. reported actual cell culture adaptation of one HEV-1 strain, JE04-1601S (HEV-1_JE04-1601S_)(Primadharsini et al., 2023). The strain was adapted to cell culture by passaging on PLC/PRF/5 and A549_1-1H8 cells and acquired multiple mutations during passaging. A cDNA clone of cell-culture adapted HEV-1_JE04-1601S_ also replicated in PLC/PRF/5 and the A549 subclone to high titers. While HEV-1_JE04-1601S_ belongs to HEV-1 subtype f, HEV-1_Sar55_ belongs to subtype b (Smith et al., 2020). Our results indicate that there could be strain-specific differences between HEV-1_JE04-1601S_ and HEV-1_Sar55,_ which should be investigated in future studies.

Following transfection of Caco-2 cells, we observed intracellular ORF2 expression as well as increasing HEV RNA titers and ORF2 protein levels in culture supernatants. However, higher RNA titers were observed for HEV-1_Sar55_ than for HEV-3_47832mc_, while higher quantities of ORF2 protein were detected for HEV-3_47832mc_ than for HEV-1_Sar55_. It has previously been shown that the majority of ORF2 protein in culture supernatants of an infected liver-derived cell line is secreted and not associated with virions (Yin et al., 2018) and we have confirmed that this is also the case in the colon-derived cell line Caco-2. Therefore, the reason for the observed discrepancy could be linked to HEV-3_47832mc_ secreting more ORF2 protein, but further studies are needed to compare the HEV-1_Sar55_ and HEV-3_47832mc_ ORF2 protein affinities of the antibodies used in the commercial antigen ELISA before drawing conclusions.

Emerson et al. have shown that transfected Caco-2 cells released HEV-1_Sar55_ virus particles that could infect the liver-derived hepatoma cell line HepG2/C3A, but the results have not been confirmed elsewhere, viral spread in HepG2/C3A cells has not been reported, and infection of Caco-2 cells has not been analyzed (Emerson et al., 2010). In our experiments, inoculation of Caco2 cells with supernatants or cell lysates from transfected Caco-2 cells enabled us to rescue replicating HEV-1_Sar55_. Caco-2 cells were sensitive to HEV-1_Sar55_ infection and supported replication to high titers. Infectious HEV-1_Sar55_ titers reached between 10^3^ to 10^4^ FFU/mL in culture supernatants, comparable to the number FFU/mL in culture supernatants of efficient HEV-3 RG systems (Meister et al., 2020; Shukla et al., 2011; Todt et al., 2020). The efficient infection of Caco-2 cells could be related to the expression of an HEV-1_Sar55_-specific receptor or entry factor, e.g. the 78-kDa glucose-regulated protein, ATP synthase subunit β, asialoglycoprotein receptor, heparan sulfate proteoglycans, integrin α3, and the EGF receptor have been shown to play a role in HEV entry (Kinast et al., 2022; Oechslin et al., 2020b; Schrader et al., 2023; Wissing et al., 2021).

Others and we observed inefficient replication of HEV-1_Sar55_ in hepatoma cell lines. On the other hand, we detected HEV-1_Sar55_ replication when Caco-2 cells were inoculated with culture supernatants of transfected PLC/PRF/5 or HuH7-Lunet BLR cells, indicating that infectious virus was released, but was unable to infect PLC/PRF/5 or HuH7-Lunet BLR cells efficiently. It is currently unclear why HEV-1_Sar55_ spreads so poorly in hepatoma cell lines and further research is required to investigate the underlying reasons. Yin et al. have previously shown that non-enveloped HEV-3 was more infectious in hepatoma cell line HepG2/C3A than quasi-enveloped HEV-3 (Yin et al., 2016). While we have not detected major differences between cell lysate- and culture supernatant-derived HEV-1_Sar55_ in Caco-2 cells, utilizing cell lysate-derived virus may improve hepatoma cell line infection.

Signs of HEV replication have previously been detected in the small intestines and colons of infected swine (Williams et al., 2001). Recently, HEV infection of primary human intestinal cells, intestinal tissue explants, and in the intestinal crypts of a chronically infected patient have also been described (Marion et al., 2020). Overall, these results indicate that intestinal cells are a site of HEV replication. It has previously been postulated that intestinal cells could become infected following uptake of HEV by the oral route and that quasi-enveloped particles released from infected intestinal cells find their way to the liver (Oechslin et al., 2020a).

Overall, our data indicate that Caco-2 cells are sensitive and permissive to HEV-1_Sar55_ infection. In future, full characterization of the model may require detection and comparison of intra- and extracellular HEV ORF2 and ORF3 protein by immunoblotting analysis. Additionally, infection of cells originating from different tissues and hosts with virus derived from intra- and extra-cellular compartments should be characterized. Nevertheless, the results presented here provide the basis for a robust and efficient RG system that enables the investigation of the full HEV-1_Sar55_ replication cycle and replication kinetics in cell culture.

## Funding acknowledgement

This study was partially funded by the Federal Ministry of Education and Research (grant number 01KI2103 “ZoRaHED”).

## CRediT authorship contribution statement

**Alexander Falkenhagen:** Writing – original draft, Methodology, Investigation, Formal analysis, Data curation, Conceptualization. **Jessica Panajotov:** Writing – review & editing, Data curation. **Reimar Johne:** Writing – review & editing, Funding acquisition, Conceptualization.

## Declaration of competing interest

The authors declare that they have no known competing financial interests or personal relationships that could have appeared to influence the work reported in this paper.

## Data Availability

Data will be made available on request.

## Appendix Supplementary materials

Image, application 1
