# Supplementary material for: Colon-derived Caco-2 cells support replication of hepatitis E virus genotype 1 strain Sar55 generated by reverse genetics
Source: Virus Res. 2024 Jun 27;347:199427. doi: 10.1016/j.virusres.2024.199427 (PMC11261143; doi:10.1016/j.virusres.2024.199427)
Supplement: Supplementary file 1 [file mmc1.docx]

Supplementary Information for

**Colon-derived Caco-2 cells support replication of hepatitis E virus genotype 1 strain Sar55 generated by reverse genetics**

Alexander Falkenhagen,^a,#^ Jessica Panajotov,^a^ and Reimar Johne^a^

^a^ Department of Biological Safety, German Federal Institute for Risk Assessment, Max-Dohrn-Str. 8-10, 10589 Berlin, Germany

^#^ Corresponding author

Address: Department of Biological Safety, German Federal Institute for Risk Assessment, Max-Dohrn-Str. 8-10, 10589 Berlin, Germany

Email address: alexander.falkenhagen@bfr.bund.de


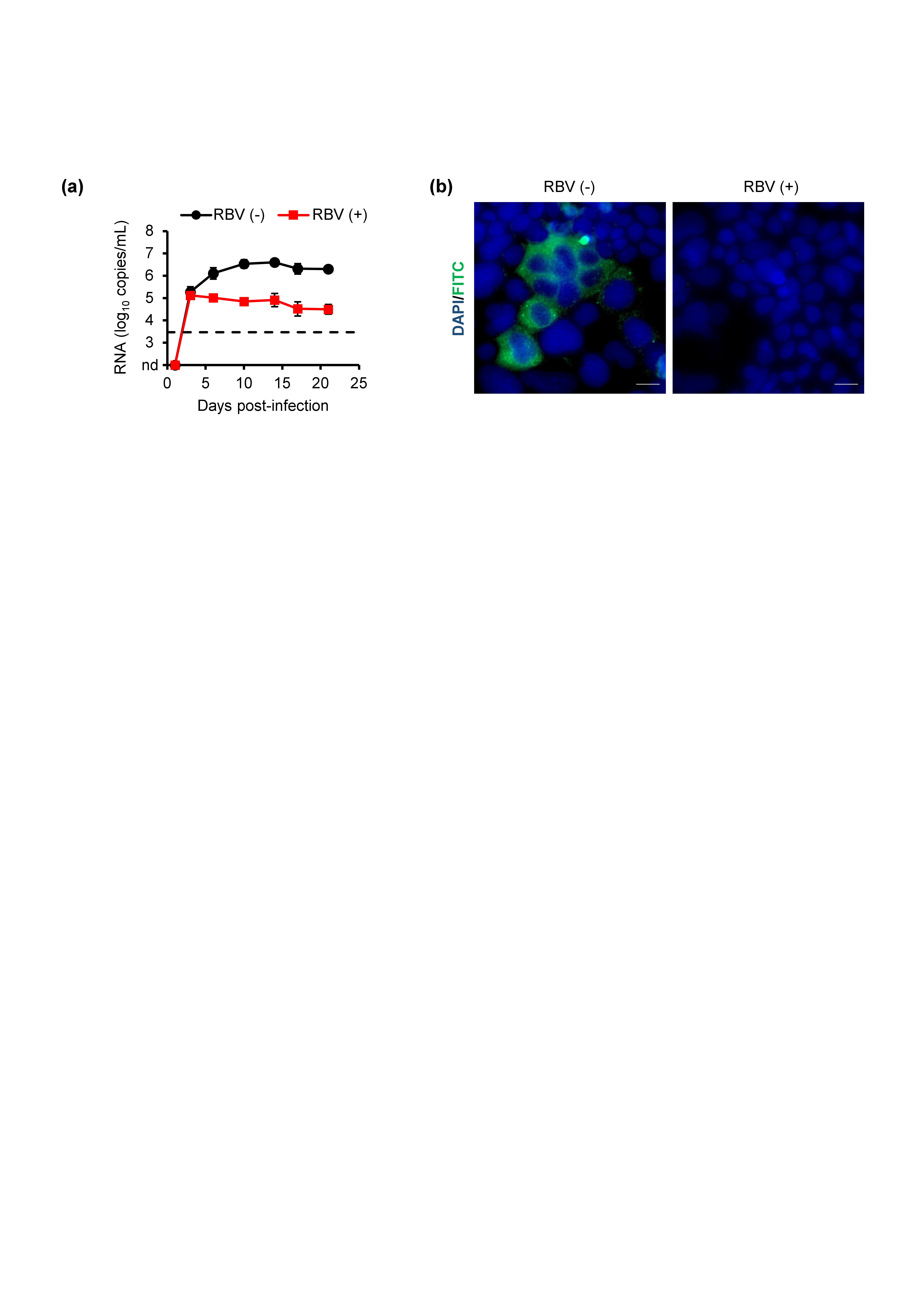


**Figure S1.** Inhibition of HEV-1_Sar55_ replication by ribavirin in Caco-2 cells. Caco-2 cells were inoculated with 1x10^7^ genome copies in the presence or absence of ribavirin. One day later, the cells were washed to remove the virus inoculum. At the indicated time points, culture supernatants were collected and fresh culture media with or without ribavirin were added to the cells. **(a)** Collected culture supernatants were analyzed for the presence of HEV RNA copies/mL by RT-qPCR in duplicates. **(b)** At 21 days post-infection, cells were analyzed by immunofluorescence microscopy for ORF2 expression. nd = not detected; RBV = ribavirin [10µM], scale bar = 20 µm.


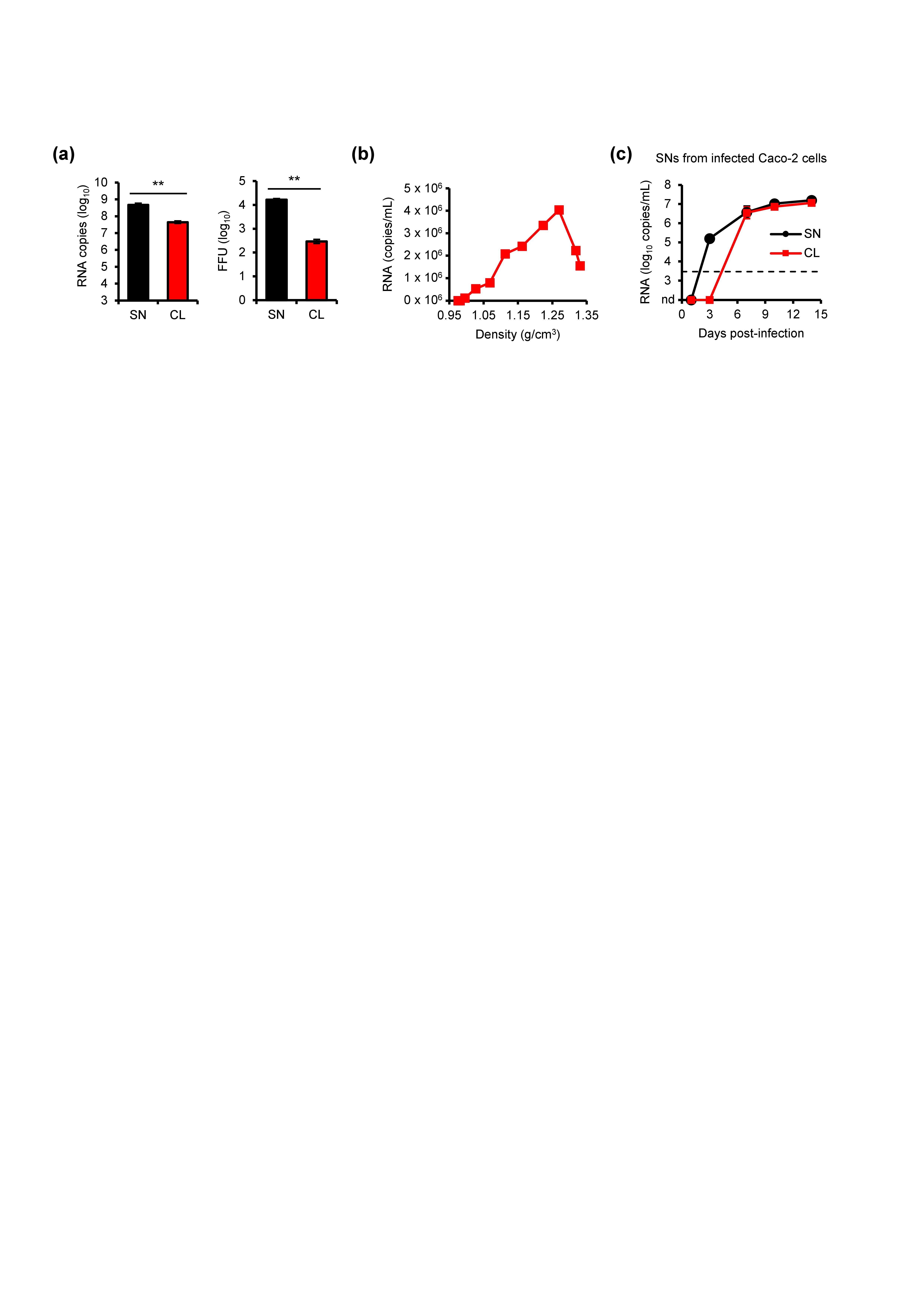


**Figure S2.** Characterization of cell lysate-derived HEV-1_Sar55_. **(a)** Caco-2 cells were transfected with HEV-1_Sar55_ RNA. On day 14 post-transfection, culture supernatants (SN) and cell lysates (CL) were collected. The total number of HEV RNA copies and the number of FFU were determined by RT-qPCR and infection of Caco-2 cells followed by immunofluorescence microscopy, respectively. HEV RNA titers are means +/− SD from two independent experiments. Infectious titers were determined for one of the experiments by titrations performed in duplicates and are expressed as means +/− SD. **(b)** HEV-1_Sar55_ derived from Caco-2 cell lysates (1x10^7^ RNA copies) were analyzed by density gradient centrifugation. The density of the different fractions was determined and the genome copy number was analyzed by RT-qPCR. **(c)** Caco-2 cells were inoculated with culture supernatant-derived (SN) or cell lysate-derived (CL) HEV-1_Sar55_ (1x10^7^ genome copies). One day later, the cells were washed to remove the virus inoculum. At the indicated time points, culture supernatants were collected and fresh culture media were added to the cells. Collected culture supernatants were analyzed for the presence of HEV RNA copies/mL by RT-qPCR. Data are means +/− SD from two independent experiments. nd = not detected; FFU = focus forming units; **p≤0.01.
